# Study protocol: a stepped wedge cluster randomised controlled trial of a healthy lifestyle intervention for people attending residential substance abuse treatment

**DOI:** 10.1186/s12889-015-1729-y

**Published:** 2015-05-03

**Authors:** Peter J Kelly, Amanda L Baker, Frank P Deane, Robin Callister, Clare E Collins, Christopher Oldmeadow, John R Attia, Camilla J Townsend, Isabella Ingram, Gerard Byrne, Carol A Keane

**Affiliations:** Illawarra Institute for Mental Health, School of Psychology, University of Wollongong, Northfields Avenue, Wollongong, 2522 Australia; School of Medicine and Public Health, Faculty of Medicine, University of Newcastle, University Drive, Callaghan, 2308 Australia; School of Biomedical Sciences and Pharmacy, Faculty of Health and Medicine and Priority Research Centre in Physical Activity and Nutrition, University of Newcastle, University Drive, Callaghan, 2308 Australia; School of Health Sciences, Faculty of Health and Medicine and Medicine and Priority Research Centre in Physical Activity and Nutrition, University of Newcastle, University Drive, Callaghan, 2308 Australia; Clinical Research Design, IT and Statistical Support Unit (CReDITSS), Hunter Medical Research Institute, Kookaburra Circuit, New Lambton Heights, 2305 Australia; School of Medicine and Public Health, Faculty of Health and Medicine, University of Newcastle, University Drive, Callaghan, 2308 Australia; Recovery Services, Australia Eastern Territory, The Salvation Army, Elizabeth Street, Sydney, 2000 Australia

## Abstract

**Background:**

Cardiovascular disease and cancer are leading causes of mortality for people with a history of alcohol or other substance use disorders. These chronic diseases share the same four primary behavioural risk factors i.e. excessive alcohol use, smoking, low intake of fruit and vegetables and physical inactivity. In addition to addressing problematic alcohol use, there is the potential for substance abuse treatment services to also address these other behaviours. Healthy Recovery is an 8-session group-based intervention that targets these multiple behavioural health risk factors and was developed specifically for people attending substance abuse treatment. This protocol describes a Cancer Institute NSW funded study that assesses the effectiveness of delivering Healthy Recovery for people who are attending residential alcohol and other substance abuse treatment.

**Methods/Design:**

The study uses a stepped wedge randomised controlled design, where randomisation occurs at the service level. Participants will be recruited from residential rehabilitation programs provided by The Australian Salvation Army. All participants who (1) currently smoke tobacco and (2) are expected to be in the residential program for the duration of the 5-week intervention will be asked to participate in the study. Those participants residing at the facilities assigned to the treatment condition will complete Healthy Recovery. The intervention is manual guided and will be delivered over a 5-week period, with participants attending 8 group sessions. All participants will continue to complete The Salvation Army residential program, a predominantly 12-step based, modified therapeutic community. Participants in the control condition will complete treatment as usual. Research staff blind to treatment allocation will complete the primary and secondary outcome assessments at baseline and then at weeks 8, 20 and 32 weeks post intervention.

**Discussion:**

This study will provide comprehensive data on the effect of delivering a healthy lifestyle intervention (i.e. Healthy Recovery) within a residential substance abuse setting. If shown to be effective, this intervention can be disseminated within other residential substance abuse programs.

**Trial registration:**

Australian and New Zealand Clinical Trials Register (ANZCTR): ACTRN12615000165583. Registered 19^th^ February 2015.

## Background

Cardiovascular disease and cancer are leading causes of mortality for people with a history of alcohol or other substance abuse problems [1,2]. The high incidence of chronic diseases is largely the result of this population engaging in multiple unhealthy lifestyle behaviours. In addition to their use of alcohol and illicit substances, people with a history of alcohol or other substance abuse problems commonly demonstrate high rates of smoking, poor dietary behaviours, and low levels of physical activity.

### Smoking

Rates of smoking are much higher for people with alcohol or other substance abuse problems compared to people in the general population [3-5]. Participants with substance abuse problems also tend to smoke substantially more cigarettes each day and demonstrate a higher rate of nicotine dependence [6]. In alcohol dependent populations, tobacco-related causes of death account for a higher rate of mortality than alcohol-related causes of death [7].

### Diet

People with a history of substance abuse problems also tend to have very poor dietary habits. Whilst they are in active addiction, malnutrition occurs in 5-30% of cases [8]. However, once they access residential treatment they are more likely to overeat, and consume energy-dense, nutrient-poor diets [4]. It is very common for people accessing treatment to report concerns with unhealthy eating patterns, unhealthy weight gain and development of obesity [9], with weight gains of approximately 3 kilograms over 12 weeks common. Both neurobiological theories of obesity [10] and interviews with recovering substance abusers [9] suggest that energy-dense diets are used as a substitute for alcohol or drug use during recovery [10].

### Physical activity

Surveys of participants in treatment settings indicate that participants have positive attitudes towards physical exercise and report the desire to increase their physical activity as a way to manage their weight gain [9]. However, less than half of all participants in treatment regularly engage in recommended levels of physical activity [4]. Exercise has been suggested as a potentially important clinical adjunct to addiction treatment, although research in this field is still in its infancy [11].

Whilst residential substance abuse treatment services provide a stable environment to target these multiple health risk behaviours, these services tend to focus almost solely on the person’s alcohol and illicit substance abuse problems. There is an opportunity for substance abuse services to also address smoking [5,12] and other risk behaviours [4] more systematically and as part of routine care.

### Multiple health behaviour change

There is “incontrovertible evidence supporting the medical and economic benefits of prevention” for cardiovascular disease and cancer [13]. It is well established that behavioural interventions focused on preventing these diseases should target smoking, poor diet, alcohol misuse and physical inactivity [14]. Although these diseases are caused by multiple health risk behaviours, historically intervention research has focused on only a single behaviour. An emerging area of preventive health research is focused on examining the effectiveness of delivering interventions that address multiple health risk behaviours (i.e. an intervention that targets both alcohol use and smoking; [15-17]). A limitation with the multiple health risk behaviour change literature is that it has focused mostly on people from the general population. It is essential that multiple health risk behaviour change interventions are developed and trialled with the most marginalised populations, as these groups tend to demonstrate the highest rates of risks (e.g. [4]), have much poorer health outcomes, and have higher rates of mortality (e.g. [1]).

There have been repeated calls to address smoking cessation [5,7], improve diet and nutrition [9], and promote physical activity in substance abuse treatment [11,18]. Yet, most residential services in Australia, and internationally, do not address these behaviours in a meaningful way [19,20]. The reluctance to address multiple health behaviours is largely the result of long standing beliefs of service providers that attempting to make too many lifestyle changes will undermine the person’s recovery from addiction. This view has particularly been expressed in terms of smoking cessation [20]. However, it is at odds with the increasing empirical support for the use of multiple behaviour change interventions [16,21]. For example, a meta-analytic review of smoking cessation interventions suggests that addressing smoking during alcohol and other substance abuse treatment actually enhances longer-term sobriety outcomes [22]. The failure to address multiple risk behaviours also neglects the significant opportunity that substance abuse services could play in helping to reduce the incidence of chronic disease for this marginalised population. Previous research has not examined the implementation of multi-focused, healthy lifestyle interventions for people with alcohol and other substance use disorders.

### The current study

The current project will be conducted at residential alcohol and other drug treatment services provided by The Salvation Army. The sites are located in New South Wales (Dooralong, Sydney, Blue Mountains) and the Australian Capital Territory (Canberra), Australia. These centres provide a modified therapeutic community that is up to 10-months in duration. The study will aim to examine the effectiveness of ‘adding’ a healthy lifestyle group program (i.e. Healthy Recovery) to treatment as usual. Adapted from the work of Baker et al. [23,24], Healthy Recovery is an 8-session group based program that aims to help participants to reduce their smoking, increase their intake of fruit and vegetables, and increase their level of physical activity. The study will be conducted as a stepped wedge randomised controlled trial, in which participants attending sites allocated to the treatment condition will complete Healthy Recovery, in addition to treatment as usual. Participants attending sites allocated to the control condition will continue to complete treatment as usual. It is expected that participants completing Healthy Recovery will demonstrate significantly lower rates of smoking, higher intake of fruit and vegetables, and higher levels of physical activity. Recruitment for the study is currently underway. The study is funded by a competitive research grant from the Cancer Institute, NSW. The University of Wollongong Human Research Ethics Committee (HE13/365) has approved the research trial, which is registered with the Australian New Zealand Clinical Trials Registry (ACTRN12615000165583).

## Methods/Design

### Study setting

The research program is being conducted at four residential alcohol and other substance abuse treatment programs: The Salvation Army William Booth House (102 beds, including 82 for males and 20 for females), The Salvation Army Blue Mountains Recovery Services (22 beds for males), The Salvation Army Canberra Recovery Services (44 beds, including 36 for males and 8 for females), and The Salvation Army Dooralong Transformation Centre (125 beds, including 85 for males and 40 for females). The treatment program across each of these sites is up to 10-months in length and is operated in the form of a modified therapeutic community. The Bridge Program aims to provide a whole of life, person-centred and coordinated care approach to recovery. Its incorporates group work, case management, one to one support, spiritual support, health care, chapel services, recreational and social activities, family involvement and employment training. The program uses a range of therapeutic approaches including motivational enhancement strategies, 12 Step Model of Recovery, Cognitive Behavioural Therapy, Case Management and Vocational Education and Training. Previous research has described these programs and examined the characteristics of people accessing these services (e.g. [4,25-28]).

### Study design

It is generally preferable to examine the effectiveness of an intervention where randomisation occurs at the participant level. However, other methods of randomisation should be used where there is the potential for contamination [29]. As the current study is being delivered within a residential group based setting, where there are high levels of interaction between participants, the likelihood of contamination is extremely high. Additionally, staff members at each of the sites will be involved in co-facilitating Healthy Recovery, making them ineligible to work with controls. Due to the small number of research sites, a cluster randomised controlled trial was not appropriate. For this reason, the proposed study will be conducted as a prospective stepped-wedge cluster randomised trial, where randomisation will occur at the service level. Stepped-wedge designs are increasingly being utilised in the evaluation of interventions within routine care [29] and are recommended where there are limited numbers of clusters [30,31].

At each step of the study design, a presentation will be made to the participants at each of the Salvation Army programs asking for participants to join the study. Participants who are interested in being involved in the study will be asked to complete a checklist confirming their eligibility to participate. The research assistant providing this presentation and supervising the completion of the eligibility checklist will be blind to the randomization sequence, and in the first and final steps will be blind to both the randomization sequence and the stepped wedge study design (i.e. different research assistants will provide these presentations to ensure blindness to the stepped wedge design). The randomization schedule will be kept centrally, with the blinded research assistant not being made aware of the randomization schedule until all participants at each site have confirmed their eligibility.

**Figure 1:**
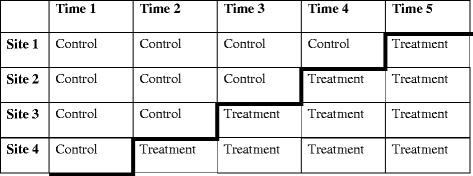
Stepped wedge randomised controlled study design. Notes. Participants in the control condition will complete treatment as usual. Participants in the treatment condition will complete Health Recovery. The order of sites will be randomly allocated.

The randomization procedures will be managed independently at the Clinical Research Design, IT, and Statistical Support Unit, University of Newcastle, NSW. Using a stepped-wedge design, each residential substance service will begin the study as a control site, providing treatment as usual to participants (Control). Sites will then progressively commence Healthy Recovery and will begin contributing to the intervention arm of the study in a stepped fashion (Treatment; see Figure 1). Independent statisticians will randomly generate the order of the sites. CONSORT procedures will be followed including using an intention to treat analysis [32].

### Participants

All participants will be attending The Salvation Army residential programs. It is expected that over the course of the study approximately 140 people will be recruited. Previous surveys of people attending The Australian Salvation Army Recovery Service Centres indicated that on average participants are 36.97 years of age [SD = 10.65; 25]. On average, alcohol is the most commonly reported primary substance of abuse (54.2%), followed by amphetamines (13.8%), cannabis (12.0%), heroin and other opiates (9.7%) and others (1.8%). All participants will complete informed written consent and all participants will be over the age of 18.

### Inclusion and exclusion criteria

All participants attending the residential program will be approached to participate in the study. Exclusion criteria have been kept to a minimum to ensure that the study can examine the effectiveness of using Healthy Recovery within a ‘real world’ setting. Participants will only be excluded from the study if: i) the person is a non-smoker, or ii) the person does not expect to be a client of the facility for the duration of the 5-week program (e.g. they are scheduled to complete the program).

### Interventions

#### Treatment condition (Healthy Recovery)

In addition to completing treatment as usual, participants in the Treatment Condition will complete Healthy Recovery. Healthy Recovery is an 8-session group based healthy lifestyle intervention that was developed for people attending residential substance abuse treatment. It is delivered over a 5-week period, with two groups being conducted each week for the first 3-weeks, and then 1 group a week conducted during the next two weeks. The first group session is completed over 90 minutes, with subsequent group sessions completed over 60 minutes. Healthy Recovery focuses on helping participants to reduce their smoking, increase their intake and variety of fruit and vegetables, and increase their level of physical activity. The intervention involves a combination of health focused psycho-education, goal setting, monitoring, motivational interviewing, and cognitive behavioural therapy. Participants are provided with pedometers to monitor their steps and these are used as a motivational tool for participants. Contingency management is also used to promote reductions in smoking. Participants will have an opportunity to earn up to $60 AUD over the course of the 5-week program if they are able to successfully reduce their smoking. Contingency management payments will be based on reductions from baseline expired carbon monoxide (CO) scores using a smokerlyzer (Micro Smokerlyzer®; Bedfont Technical Instruments Ltd, Kent, UK). In weeks 2 and 3 participants will have an opportunity to earn $5 AUD for any reductions in their CO score, $7 for halving their CO score and $15 for demonstrating that they have quit smoking. In weeks 4 and 5 participants are only rewarded for halving their CO score or quitting smoking. If smoking at least 10 cigarettes per day, participants are recommended to commence nicotine replacement therapy (NRT). If required, the NRT will be prescribed to participants by the general practitioner that attends the treatment program. Costs associated with NRT are subsidised under the Australian Pharmaceutical Benefits Scheme. The group sessions will be co-facilitated by a Salvation Army drug and alcohol worker and a member of the research team (i.e. Intern Psychologist or Clinical Psychologist). To track adherence to the intervention, group attendance logs are maintained (participants are trained to complete these logs during groups sessions). Participants are also asked to log their health behaviours each day onto a worksheet (i.e. number of cigarettes, servings of fruit and vegetables, steps walked, minutes of exercise). These worksheets are photocopied by the research staff and kept to monitor adherence.

#### Control condition - treatment as usual (TAU)

Participants in the Control Condition will continue to complete treatment as usual (i.e. The Salvation Army residential substance abuse program).

#### Treatment fidelity

All intervention sessions will be audiotaped. Independent psychologists will rate a random allocation of 20% of treatment sessions for fidelity and competence.

### Outcome measures

A limitation with previous stepped wedge randomised controlled trials is that outcome assessment officers have not been blind to the treatment condition [33]. To ensure the blind measurement of the primary and secondary assessment measures, a combination of assessment officers will be involved in the study. Non-blind assessment officers will complete the face-to-face baseline assessments. This assessment will include administering the Structured Clinical Interview of the DSM 5-CT to identify 12-month and lifetime history of alcohol or other substance abuse and dependence disorders. The Timeline Follow-back and Opioid Treatment Index will be completed to examine use of alcohol and other substances before attending The Salvation Army program (OTI; [34]), ([35,36]). The baseline face-to-face assessment will also involve collecting demographic characteristics, physical health history and mental health history (see Table 1).

**Table 1:** **Assessment instruments used for the current study**

**Domain assessment and instrument used**	**Week 1**	**Weeks 3 to 7**	**Week 8**	**Weeks 20, 32**
**Background and descriptive**				
Demographic information	✓			
Medical history (e.g. previous diagnosis of chronic diseases, family history of chronic diseases)	✓			
Mental health history (e.g. previous treatment, previous diagnosis, use of medication)	✓			
**Physical health**				
Body Mass Index (height and weight)	✓		✓	
Waist circumference	✓		✓	
Expired carbon monoxide	✓	✓	✓	
Blood pressure	✓		✓	
**Physical activity**				
International physical activity questionnaire	✓		✓	✓
Accelerometer	✓		✓	
Importance and confidence to increase physical activity	✓		✓	✓
**Diet**				
Healthy Eating Quiz	✓		✓	✓
Average servings of both fruit and vegetables over the previous 2-weeks	✓		✓	✓
Importance and confidence to improve diet	✓		✓	✓
**Alcohol and substance abuse measures**				
Structured Clinical Interview for DSM Disorders: Alcohol and Substance Use sections only	✓			
Addiction Severity Index: Alcohol and Drug composite scores	✓		✓	✓
Opiate Treatment Index	✓		✓	✓
Fagerstrom test for nicotine dependence	✓		✓	✓
7-day point prevalence of smoking	✓		✓	✓
Use of nicotine replacement therapy and number of quit attempts	✓		✓	✓
Smoking stages of change (Short Form)	✓		✓	✓
Importance and confidence to quit smoking	✓		✓	✓
**Mental health measures**				
Addiction Severity Index: Mental Health composite score	✓			
Quality of Life	✓		✓	✓

The non-blind assessment officers will also collect biomedical measures from participants who are attending the residential program at baseline and 8-weeks. This will include height, weight, blood pressure, waist circumference and expired carbon monoxide. Participants will also be asked to wear accelerometers (ActiGraph GT3X+) for a 5-day wear period from pre-midday Monday through to pre-midday Friday. This will ensure three full days of accelerometer wear (≥10 hours/day) data are collected – the minimum requirement deemed acceptable for analysis [37,38]. Each participant will be fitted with an accelerometer device, positioned over the non-dominant hip, affixed with an elastic band around the waist. Participants will be instructed to wear the device at all times except when bathing.

### Primary and secondary outcome measures

Independent assessment officers, blind to both the treatment condition and the design of the study, will conduct telephone interviews to complete the primary and secondary outcome assessment measures throughout the study. This will help to reduce bias associated with this type of study (e.g. participants are aware of their allocated treatment condition). Outcome measures will be collected at baseline, following the intervention (8-weeks) and then at 20 weeks and 32 weeks (3- and 6-months) follow-up. Each participant will be offered $20 AUD for completion of the baseline assessment and each subsequent follow-up assessment, as reimbursement for the time associated with completing the measures. For assessments completed while a participant is a resident at one of The Salvation Army programs, he or she will be remunerated in cash [39]. Due to the difficulties associated with posting cash payments, gift certificates will be posted to those participants who complete follow-up assessments once they leave the residential program.

The primary outcome measure will be the number of cigarettes smoked per day. Smoking reduction was selected as the primary dependent variable as this has been shown to be an important clinical strategy when working with long-term, heavy smokers towards smoking cessation. In a review of the smoking reduction literature, it was concluded that “smoking reduction increases the probability of later quitting” [40]. Smoking reduction is also likely to be associated with health and financial benefits for participants.

The secondary outcome measures will examine smoking, diet, physical activity and substance use. *Smoking -* 7-day smoking point prevalence abstinence self reported by participants. For participants who are still attending the residential program, abstinence will be verified by expired carbon monoxide tests (conducted by non-blind assessment officers). Participants will also be asked to report the number and duration of quit attempts since the last assessment time point, and readiness to quit smoking as measured by the Smoking Stages of Change (Short Form; [41]). Importance of and confidence in quitting smoking will be measured with two questions requiring ratings from 0 to 10: (1) How important is it to you personally to quit smoking?; (2) If you decided right now to quit smoking, how confident do you feel about succeeding with this? These scales have been adapted from motivational interviewing [42] and previously used to assess a variety of health behaviours (e.g. [43]). *Diet* – Participants will be asked to report the number of serves of fruit and vegetables that they usually eat each day, over the last 2-weeks. Fruit and vegetable consumption, as well as overall diet quality will also be assessed by the Australian Recommended Food Score (ARFS) index [44]. The ARFS uses a subsample of questions from the Australian Eating Survey Food Frequency Questionnaire [45] to assess adherence to eating patterns recommended in the Australian Dietary Guidelines for adults [46]. Importance of and confidence in improving diet were assessed with two questions requiring ratings from 0 to 10: (1) How important is it to you to improve your diet?, (2) If you decided right now to improve your diet, how confident do you feel about succeeding with this? *Physical activity* – The International Physical Activity Questionnaire (IPAQ) will be used to assess the participant’s self-reported amount of vigorous and moderate physical activity [47]. The IPAQ will also examine the amount of walking, as well as the amount of sedentary behaviour in the previous week. Importance of and confidence in increasing physical activity will also be assessed with two questions requiring ratings from 0 to 10: (1) How important is it to you to improve your physical activity?, (2) If you decided right now to improve your physical activity, how confident do you feel about succeeding with this?

### Data analysis

#### Power analysis

The primary outcome is number of cigarettes/day at 8 weeks follow-up. Unpublished pilot data suggests this has a SD of 12 cigarettes/day. Assuming a baseline/follow-up correlation of 0.25 we would need a sample of 85 before and 85 after intervention to give the study 80% power to detect a 5 cigarette/day intervention effect at a 5% significance level. We have added another 15% to allow for non-parametric distribution of the data, i.e. 98 people before and after. To account for loss to follow-up we have added another 40% (based on previous RCTs conducted by the researchers), providing a sample of about 140 people who will complete before and after measures.

### Analysis plan

Since the number of clusters is small, we will use a fixed effects linear regression model to assess the effect of the intervention. The outcome variable will be number of cigarettes/day at 8 weeks, 20-weeks and 32-weeks. Baseline number of cigarettes/day will be included as a covariate. We will include a fixed effect for treatment centre, which accounts for differences in the average level of the outcome across centres and the association between before/after and centre induced by the stepped-wedge design. The main predictor of interest will be a before/after intervention variable, which would be at a different time point for each centre and will measure the intervention effect, which after adjusting for centre is a weighted average of the effect within each centre. Time will be included in the model as a continuous measure to account for any secular trends. We will use boot-strapped estimates of the standard error to account for any violations in the assumption of normality of residuals. The data will be analysed following the intent-to-treat principle. The primary analysis will be a complete case analysis, with sensitivity analysis undertaken to include all participants under an appropriate model for missing data such as multiple imputation. Analysis will be conducted independently by statisticians in the Clinical Research Design, IT and Statistical Support Unit, University of Newcastle.

## Discussion

It is common for people accessing substance abuse treatment to smoke cigarettes, have poor dietary habits and engage in low levels of physical activity [4]. In conjunction with their extensive history of alcohol and other substance abuse, these unhealthy behaviours increase the risk of this population developing cardiovascular disease, cancer and other lifestyle related chronic diseases. There is an opportunity for substance abuse treatment providers to address smoking and other health behaviours as part of routine care. The present study aims to examine the effectiveness of ‘adding’ Healthy Recovery, a group based healthy lifestyle intervention, to residential alcohol and other substance abuse treatment. It is expected that when compared to the control condition, participants completing Healthy Recovery will demonstrate greater reductions in the number of cigarettes that they smoke each day. It is also expected that there will be greater increases in their intake and variety of fruit and vegetables, overall diet quality and their level of physical activity. As the current study is the first randomised controlled trial of a healthy lifestyle intervention within residential substance abuse populations, the results potentially hold important implications for the way that multiple health risk behaviour change interventions are delivered across a range of substance use treatment settings.

### Strengths and limitations

The current study is being conducted across The Salvation Army residential substance abuse treatment programs. The strength of conducting this type of ‘real world’ research is that it is more representative of actual clinical practice and helps to provide some evidence regarding the feasibility of using these types of interventions as part of ongoing routine care. The research design also includes additional attempts to increase the generalisability of the results by using very inclusive eligibility criteria.

Another strength of the study is that all of the primary and secondary outcome measures used in the study will be collected by assessment officers who are both blind to treatment condition and to the design of the study. Ensuring the blindness of assessment officers to treatment condition has been an issue that has plagued stepped wedge designs. For example, a systematic review examining studies that have used stepped wedge designs found that the articles reported that assessment officers were not blind to treatment condition or they did not specify adequately if assessment officers were blind to condition [33]. The major difficulty with ensuring blindness in stepped wedge designs is that once a study is allocated to the treatment condition, research staff visiting those sites are aware that that site will continue to be allocated to the treatment condition. In the current study we have made the decision to have two sets of assessment officers. One team of non-blind assessment officers visit the residential programs and conduct face-to-face assessments. The primary purpose of these assessment officers is to build a relationship with the participants, introduce the participants to the blind assessment officers over the phone, and to collect background information. The second set of assessment officers, blind to treatment condition and the study design, will be based at the University of Wollongong. We believe that the combination of blind and non-blind assessment officers will help ensure that we maintain a strong relationship with the participants (i.e. promoting follow-up rates) and maintain the scientific credibility of the study.

As with other studies conducted in residential substance abuse treatment settings, a significant challenge is the high rate of unplanned dropout from these units. About 57% of people prematurely leave The Salvation Army programs within the first 3-months of treatment [25], with similar proportions also being reported across the broader literature (see [48]). To help address this concern, Healthy Recovery is delivered over a 5-week period, to maximise the number of groups that participants will complete. A further challenge for the current study will be retaining participants at follow-up. People with alcohol and other substance abuse disorders are traditionally very difficult to follow-up. This is further complicated with residential facilities, as participants often move outside of their local area to attend treatment. Attempts to improve follow-up rates in the current study will include using telephone follow-up, obtaining contact details of significant others to help with locating participants, reinforcing to participants the importance of conducting follow-up and financially compensating participants for the time required to complete the assessments (AUD$20).

## Conclusion

The current study will be the first randomised controlled trial of a healthy lifestyle intervention with a residential substance abuse population. The study seeks to address a significant gap in the multiple health behaviour change literature by examining the effectiveness of implementing an 8-session healthy lifestyle group program within residential substance abuse treatment programs. It is expected that, when compared to the control condition, participants completing Healthy Recovery will demonstrate greater reductions in the number of cigarettes that they smoke, greater increases in fruit and vegetable consumption, and greater increases in the amount of physical activity they engage in. It is anticipated that results from the current study will also help to inform the development and implementation of healthy lifestyle interventions for other marginalized populations that tend to share the same characteristics as people accessing substance abuse treatment services (i.e. mental illness, poverty, homelessness, criminal involvement).
